# Estrogen-Mediated Upregulation of Noxa Is Associated with Cell Cycle Progression in Estrogen Receptor-Positive Breast Cancer Cells

**DOI:** 10.1371/journal.pone.0029466

**Published:** 2011-12-22

**Authors:** Wensheng Liu, Wendy M. Swetzig, Rajesh Medisetty, Gokul M. Das

**Affiliations:** 1 Department of Pediatrics, The State University of New York, University at Buffalo, Buffalo, New York, United States of America; 2 Center for Genetics and Pharmacology, Department of Pharmacology and Therapeutics, Roswell Park Cancer Institute, Buffalo, New York, United States of America; 3 Dr. Reddy's Laboratories, Ltd., Department of Biologics, Hyderabad, Andhra Pradesh, India; 4 Center for Genetics and Pharmacology, Department of Pharmacology and Therapeutics, Roswell Park Cancer Institute, Buffalo, New York, United States of America; Florida International University, United States of America

## Abstract

Noxa is a Bcl-2-homology domain (BH3)-only protein reported to be a proapoptotic member of the Bcl-2 family. Estrogen has been well documented to stimulate cell growth and inhibit apoptosis in estrogen receptor (ER)-positive breast cancer cells. Intriguingly, recent reports have shown that 17β-estradiol (E2) induces Noxa expression, although the mechanisms underlying E2-mediated induction of Noxa and its functional significance are unknown. Using MCF7 human breast cancer cells as an experimental model, we show that Noxa is upregulated by E2 via p53-independent processes that involve c-Myc and ERα. Experiments using small interfering ribonucleic acids (siRNA) to specifically knock down p53, c-Myc, and ERα demonstrated that c-Myc and ERα, but not p53, are involved in the transcriptional upregulation of Noxa following E2 treatment. Furthermore, while E2 promoted the recruitment of c-Myc and ERα to the *NOXA* promoter in chromatin immunoprecipitation (ChIP) assays, E2 did not induce p53 recruitment. Interestingly, E2-mediated upregulation of Noxa was not associated with apoptosis. However, siRNA-mediated knockdown of Noxa resulted in cell cycle arrest in G_0_/G_1_-phase and significantly delayed the G_1_-to-S-phase transition following E2 treatment, indicating that Noxa expression is required for cell cycle progression in ER-positive breast cancer cells.

## Introduction

Noxa/Phorbol 12-myristate 13-acetate (PMA)-Induced Protein 1 (PMAIP1)/Adult T-cell Leukemia-derived PMA-responsive (APR) is a proapoptotic Bcl-2-homology domain 3 (BH3)-only member of the Bcl-2 family of proteins [1]. The Bcl-2 family of proteins is subdivided into three different classes, according to conservation of the Bcl-2 homology (BH) domains, BH1-4 [2]–[6]. The first class consists of the multi-domain prosurvival proteins, which include Bcl-2, Bcl-xL, Mcl-1, Bcl-w/BCL2L2, Bfl-1/A1, and Bcl-B/Bcl2L10; the second class consists of the multi-domain proapoptotic proteins, which include Bax, Bak, and Bok/Mtd; the third class consists of the BH3-only proapoptotic proteins, which include Noxa, Puma, Bid, Bad, Bim, Bik, Bmf, and Hrk. [2]–[7]. Various combinations of these three classes of Bcl-2 proteins come together to form heterodimeric complexes at the mitochondria, resulting in the induction or suppression of apoptosis. While the BH3-only proteins Puma, Bid, and Bim can induce apoptosis by directly interacting with and activating the multidomain proapoptotic members (such as Bax and Bak), Noxa induces apoptosis by suppressing prosurvival Mcl-1 [8]–[11]. Under normal cellular conditions, proapoptotic Bak is maintained as a heterodimer with prosurvival Mcl-1; however, in response to various cellular stresses, Noxa becomes upregulated and competes with Bak for binding to Mcl-1, thereby releasing Bak from prosurvival Mcl-1 and initiating Bak-mediated apoptosis [8]–[10], [12], [13].

Recent studies have shown that Noxa plays important roles in many physiological processes other than apoptosis. In human ovarian surface epithelial cells, Noxa is required for Ras-induced autophagy [14]. In Bcl-2 overexpressing MCF7 cells, cisplatin-induced Noxa expression is required for lipid peroxidation [15]. Furthermore, some studies suggest that Noxa may play a pro-survival role under certain contexts. In acute lymphoblastic leukemia cells, Noxa is repressed during glucocorticoid-induced apoptosis [16], and Noxa also promotes cell growth by stimulating glucose consumption via the pentose phosphate pathway [17], [18]. These data highlight the multiple roles of Noxa as a context-dependent regulator of many different physiological processes, including, but not limited to, apoptosis.

Although Noxa is traditionally known to be a transcriptional target gene of tumor suppressor p53 due to its well-defined role in p53-mediated apoptosis [1], [2], [5], [19]–[21], many p53-independent mechanisms of Noxa upregulation have been identified, also. For example, the transcription factors c-Myc [22], Hypoxia-Inducible Factor (HIF)-1α [23], cAMP Response Element Binding Protein (CREB) [24] and E2F Transcription Factor 1 (E2F1) [25] have been described to mediate p53-independent transcription of Noxa. Furthermore, recent studies have shown that 17β-estradiol (E2) induces Noxa expression in breast cancer cells [26], [27], although the mechanisms underlying E2-mediated induction of Noxa have not been reported. Notably, E2 is well-documented to stimulate cell growth and promote cell cycle progression in estrogen receptor (ER)-positive breast tumors [28]–[30]. As the majority of breast cancers are initially hormone-dependent [31], [32], E2-mediated upregulation of Noxa expression could be of particular relevance to breast tumor biology. However, the functional significance of E2-mediated upregulation of Noxa in breast cancer cells has not been thoroughly studied, and the relationship between E2-dependent induction of Noxa and E2-dependent stimulation of cell growth remains to be elucidated.

Here we report that E2 induces Noxa expression via p53-independent pathways that are mediated by c-Myc, ERα, and E2F1/RB. For the first time, we show that knocking down Noxa inhibits E2-induced cell cycle progression in breast cancer cells, suggesting a novel role for Noxa as a cell cycle regulator in ER-positive breast tumors.

## Results

### c-Myc mediates E2-induced Noxa transcription in human breast cancer cells

**Figure 1:**
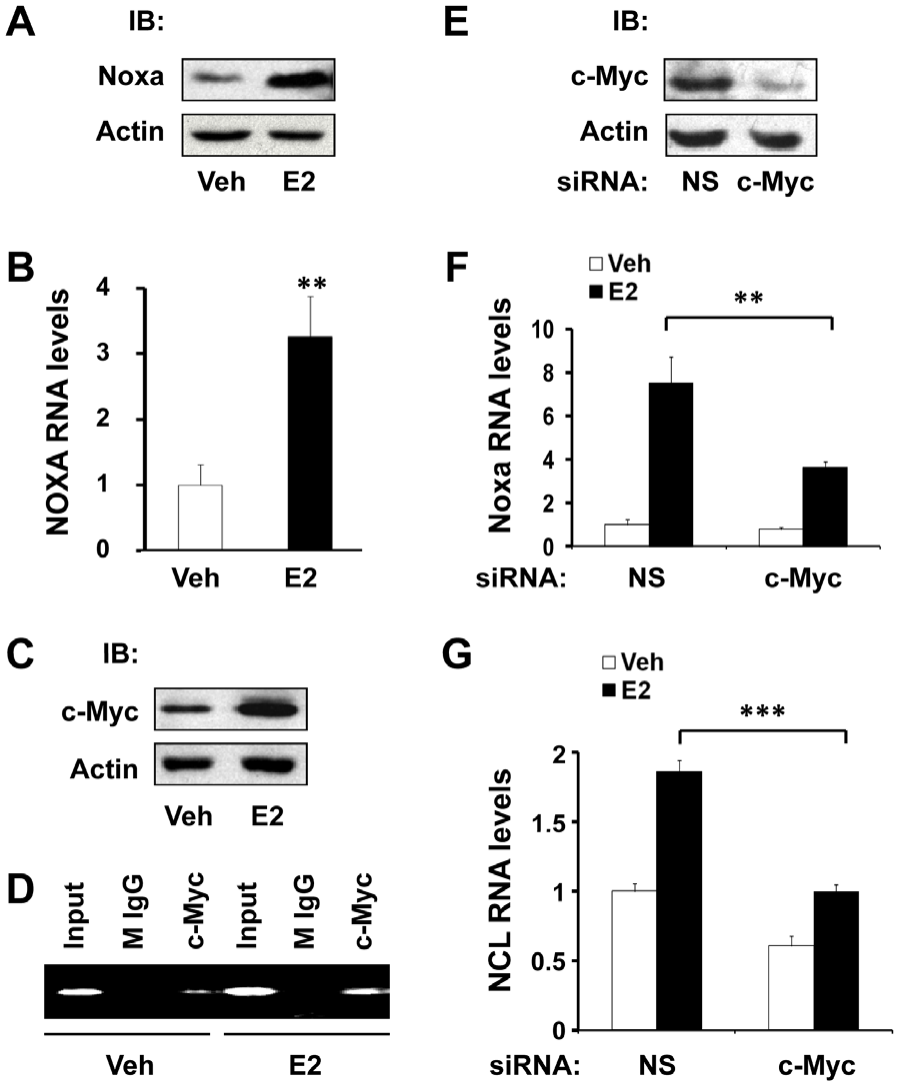
c-Myc mediates E2-induced Noxa transcription in human breast cancer cells. (**A–C**) MCF7 cells were treated with vehicle (veh) or E2 (10 nM) for 16 hr and then harvested for analysis of Noxa protein expression by western blotting (A), Noxa mRNA expression by qPCR (B), and c-Myc protein expression by western blotting (C). (**D**) MCF7 cells were treated with vehicle (veh) or E2 for 4 hr, and ChIP assays using anti-c-Myc antibody or normal mouse IgG (M IgG, antibody control) were performed to analyze the effect of E2 on the recruitment of c-Myc to the *NOXA* promoter. Immunoprecipitated ChIP DNA was analyzed by PCR using site-specific primers that amplify a region of the *NOXA* promoter that contains a c-Myc binding site at +85 bp. (**E**) MCF7 cells were transfected with non-silencing control (NS) or c-Myc siRNA for 48 hr and then harvested for analysis of c-Myc protein levels by western blotting. (**F, G**) MCF7 cells were transfected with non-silencing control (NS) or c-Myc siRNA for 24 hr, followed by treatment with vehicle (veh) or E2 (10 nM) for 8 hr, and the relative mRNA expression levels of Noxa (F) and Ncl (G) were analyzed by qPCR. Graphical data points in B, F, and G are means ± S.D. of three independent experiments (** *P*<0.01, *** *P*<0.001).

It has been reported that Noxa is upregulated in response to E2 treatment in breast cancer cells [26], [27], and Noxa expression is co-clustered with ERα expression in breast tumors [27]. Consistent with these previous reports, we found that Noxa protein and mRNA were upregulated in response to E2 treatment in estrogen-responsive MCF7 human breast cancer cells (Fig. 1A & 1B). It has been reported that *NOXA* is a transcriptional target gene of c-Myc [22] and that *MYC* is a transcriptional target gene of E2 [33]. Therefore, we tested if E2-dependent induction of Noxa is mediated by c-Myc. E2 treatment induced c-Myc expression (Fig. 1C) and increased the amount of c-Myc protein that was bound to the *NOXA* promoter (Fig. 1D), as assayed by chromatin immunoprecipitation (ChIP) assays. Transient transfection of c-Myc small interfering RNA (siRNA) substantially knocked down c-Myc protein expression (Fig. 1E) and partially blocked E2-dependent induction of Noxa (Fig. 1F). As a positive control, knocking down c-Myc also blocked E2-dependent induction of nucleolin (NCL) (Fig. 1G), which was previously reported to be an E2-responsive transcriptional target gene of c-Myc [34]. These data collectively indicate that E2 induces Noxa expression via a mechanism that is partially dependent upon c-Myc; however, additional mechanisms likely exist, as loss of c-Myc did not completely abolish E2's ability to induce Noxa expression (Fig. 1F).

### E2-induced Noxa transcription is ERα-dependent and p53-independent

**Figure 2:**
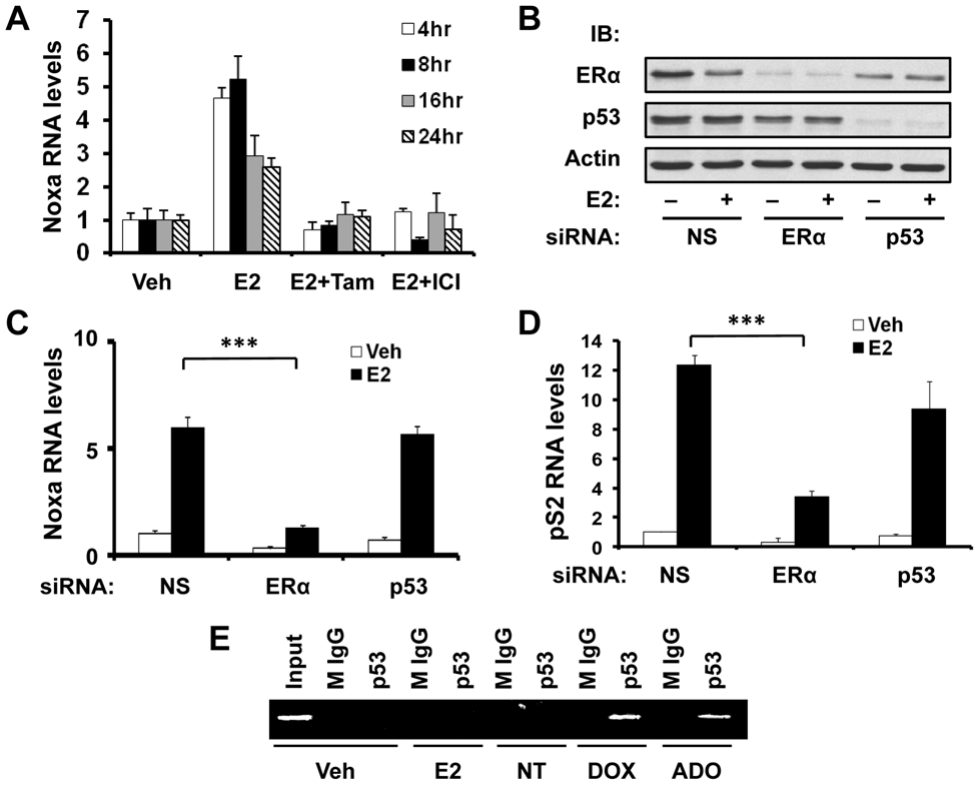
E2-induced Noxa transcription is ERα-dependent and p53-independent. (**A**) MCF7 cells were treated with vehicle (veh), 10 nM E2 alone, 10 nM E2 in combination with 1 µM tamoxifen (Tam), or 10 nM E2 in combination with 1 µM ICI 182780 (ICI) for various times. Four, 8, 16 and 24 hr after treatment, cells were harvested, and Noxa mRNA expression levels were analyzed by qPCR. (**B, C, & D**) MCF7 cells were transfected with non-silencing control (NS), ERα, or p53 siRNA for 24 hr, followed by treatment with vehicle (veh) or E2 (10 nM) for 8 hr, and then harvested for analyses of ERα, p53, and β-actin (internal control) protein expression by western blotting (B), Noxa mRNA expression by qPCR (C), and pS2 mRNA expression by qPCR (D). (**E**) MCF7 cells were treated with vehicle (veh), E2 (10 nM), doxorubicin (DOX; 1.70 µM), adozelesin (ADO; 4 nM), or left without treatment (NT) for 16 hr. Occupancy of p53 on the proximal region of the *NOXA* promoter was determined by ChIP assays, using anti-p53 antibody or normal mouse IgG (M IgG; control antibody) for immunoprecipitation. Graphical data points in A, C, and D are means ± S.D. of three independent experiments (*** *P*<0.001).

Since the primary mechanism of action of E2 is elicited through ERα, we tested if ERα contributed to E2-induced Noxa expression, in addition to c-Myc. Indeed, analysis of Noxa mRNA levels in MCF7 cells showed that the antiestrogens tamoxifen and ICI 182780 (fulvestrant) antagonized E2-induced upregulation of Noxa (Fig. 2A), suggesting the involvement of ERα. Furthermore, when ERα was knocked down (Fig. 2B), E2-induced Noxa expression was drastically reduced (Fig. 2C), demonstrating that E2-induced Noxa expression is ERα-dependent. As a positive control, E2-dependent induction of *pS2 (TFF1)*, a prototypic transcriptional target gene of ERα, was also reduced when ERα was knocked down (Fig. 2D). Notably, although Noxa expression is highly regulated by p53, E2-dependent induction of Noxa expression appears to be a p53-independent process because transient knock down of p53 (Fig. 2B) did not affect the ability of E2 to induce Noxa expression (Fig. 2C). Consistent with this result, E2 treatment did not result in the recruitment of p53 to the *NOXA* promoter, as compared to treatment with the DNA damaging agents doxorubicin and adozelesin (Fig. 2E), which were used as positive controls. These data indicate that under conditions that do not cause genomic damage, ERα regulates Noxa expression via a p53-independent process.

### E2F1 mediates ERα-binding to the NOXA promoter in the presence of E2

**Figure 3:**
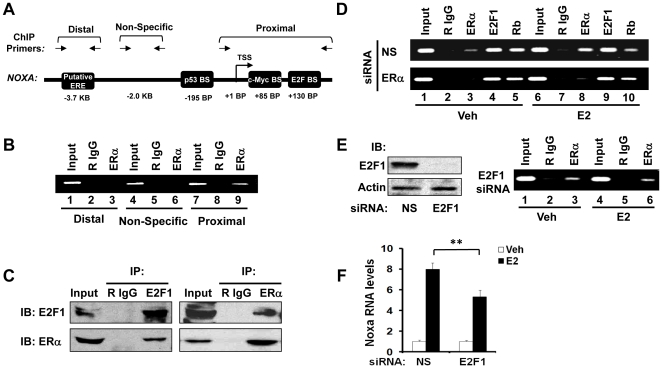
E2F1 mediates ERα-binding to the NOXA promoter in the presence of E2. (**A**) Schematic representation of the *NOXA* promoter. Horizontal arrows indicate the primers used for PCR in site-specific ChIP assays. Note that the figure is not drawn to scale. BS: binding site. TSS: transcription start site. ERE: estrogen response element. KB: distance in kilobases from the TSS. BP: distance in base pairs from the TSS. (**B**) ChIP assays were performed on MCF7 cell lysates to detect ERα binding to the *NOXA* promoter. Anti-ERα antibody or normal rabbit IgG (R IgG; control antibody) were used to immunoprecipitate ChIP DNA, and ChIP PCR was performed using primers that amplify a putative ERE site at −3.7 kb (lanes 1–3), a non-specific (NS) negative control site where no binding is expected to occur (lanes 4–6), and the proximal region of the *NOXA* promoter (lanes 7–9). PCR products were resolved on agarose gels. (**C**) Co-immunoprecipitation (co-IP) of protein complexes containing ERα and E2F1. Left panel: normal rabbit IgG (R IgG; antibody control) or anti-E2F1 antibody was used for immunoprecipitation (IP) and anti-E2F1 and anti-ERα antibodies were used for immunoblotting (IB). Right panel: R IgG or anti-ERα antibody was used for IP and anti-ERα and anti-EF21 antibodies were used for IB. (**D**) MCF7 cells were transfected with non-silencing control (NS) or ERα siRNA for 24 hr, followed by treatment with E2 (10 nM) or vehicle (veh) for 4 hr. The recruitment of ERα, E2F1, and RB to the proximal region of the *NOXA* promoter was analyzed by ChIP assays, as in B. (**E**) Left panel: MCF7 cells were transfected with non-silencing control (NS) siRNA or E2F1 siRNA for 48 hr, and E2F1 protein levels were monitored by western blotting. Right panel: MCF7 cells were transfected with E2F1 siRNA for 24 hr and then treated with vehicle (veh) or E2 (10 nM) for 4 hr. Recruitment of ERα to the proximal region of the *NOXA* promoter was analyzed by ChIP assays, as in B. (**F**) MCF7 cells were transfected with non-silencing control (NS) siRNA or E2F1 siRNA for 24 hr and then treated with vehicle (veh) or E2 (10 nM) for 8 hr. Noxa mRNA expression was assayed by qPCR. Graphical data points in F are means ± S.D. of three independent experiments (** *P*<0.01).

After determining that ERα is a mediator of E2-dependent induction of Noxa, we next tested if *NOXA* is a direct transcriptional target of ERα. We identified a putative estrogen response element (ERE) within the *NOXA* promoter at −3.7 Kb (Fig. 3A) by using the ‘Dragon ERE Finder’ program [35]. However, instead of binding to this putative ERE site, we found that ERα was recruited to the proximal region of the *NOXA* promoter in ChIP assays (Fig. 3B, compare lanes 3 & 9). Since no ERE was identified in the proximal region of the *NOXA* promoter, we hypothesized that ERα was accessing the DNA by tethering onto other proteins. It has been reported that the murine *NOXA* gene is a transcriptional target of E2F1 [25], and a potential E2F binding site has been identified within the human *NOXA* promoter [22], near the site at which we observed ERα binding (Fig. 3A). Therefore, we tested if ERα was able to bind to E2F1 protein directly, which would suggest that ERα might access the *NOXA* promoter by tethering to DNA-bound E2F1. The results of co-immunoprecipitation experiments showed that ERα and E2F1 coexisted in a single protein complex in MCF7 cells (Fig. 3C). Consistent with these data, site-specific ChIP assays showed that both E2F1 and its partner, the retinoblastoma tumor suppressor protein (RB), were present on the region of the *NOXA* promoter to which ERα binds (Fig. 3D, upper panel). E2 treatment augmented ERα binding but decreased RB binding to the *NOXA* promoter (Fig. 3D, upper panel, compare lanes 3 & 8 and 5 & 10); however, the binding of RB to the *NOXA* promoter was not affected by E2 when ERα was knocked down (Fig. 3D, compare lanes 5 & 10 of upper panel to lanes 5 & 10 of lower panel). These data indicate that in the absence of E2, E2F1 recruits RB to the *NOXA* promoter, whereas in the presence of E2, E2F1 recruits ERα on the *NOXA* promoter. This inverse correlation between ERα and RB binding to the *NOXA* promoter in the presence of E2 suggests that ERα and RB may be recruited to the *NOXA* promoter via E2F1 in mutually exclusive manners, although this remains to be formally tested. In support of the hypothesis that E2F1 is required for E2 to be able to increase the amount of ERα that is recruited to the *NOXA* promoter, we found that when E2F1 was knocked down (Fig. 3E, left panel), E2 was unable to increase ERα recruitment (compare lanes 3 & 6 of Fig. 3E, right panel, to lanes 3 & 8 of Fig. 3D, upper panel). As a consequence, the ability of E2 to induce Noxa expression was reduced when E2F1 was knocked down (Fig. 3F). Together, these data suggest that ERα is recruited to the *NOXA* promoter by E2F1 and that E2F1 is required for ERα to mediate upregulation of Noxa in response to E2 treatment.

### Noxa knock-down arrests cells in G_1_/G_0_-phase and delays E2-induced S-phase entry

**Figure 4:**
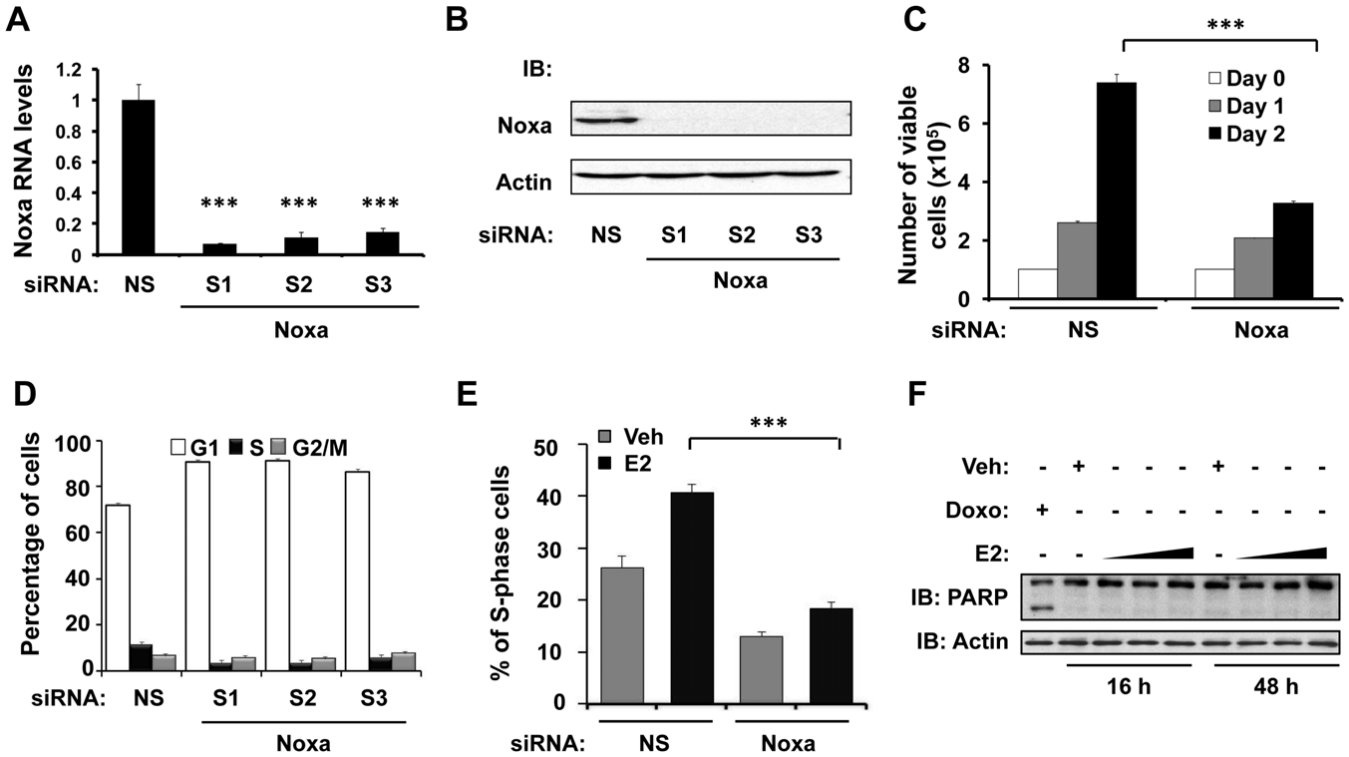
Noxa knock-down arrests cells in G_1_/G_0_ phase and delays E2-induced S-phase entry. (**A & B**) MCF7 cells were transfected with 50 nM of non-silencing control (NS) siRNA or Noxa (S1, S2, and S3) siRNA for 24 hr, after which Noxa mRNA expression was analyzed by qPCR (A), and Noxa protein expression was analyzed by western blotting (B). S1, S2 and S3 are three different siRNA sequences that target different regions of Noxa mRNA. (**C**) MCF7 cells were transfected with non-silencing control (NS) siRNA or Noxa siRNA (a pool of S1, S2, and S3) for 0, 1, or 2 days, as indicated. The number of viable cells was determined using the “Cell Titer-Glo Assay” kit. (**D**) MCF7 cells were transfected with 50 nM of non-silencing control (NS) siRNA or Noxa siRNA (a pool of S1, S2, and S3) for 48 hr. Cell cycle distribution was analyzed by flow cytometry using the PI-staining method. (**E**) MCF7 cells were transfected with non-silencing control siRNA (NS) or Noxa siRNA (a pool of S1, S2, and S3) for 32 hr, followed by E2 (10 nM) or vehicle (veh) treatment for 16 hr. The S-phase population was analyzed by flow cytometry using the PI-staining method. (**F**) Dose titration and timecourse experiments were performed to determine the effect of E2-induced Noxa expression on apoptosis, using doxorubicin treatment as a positive control. MCF7 cells were treated with vehicle (Veh), 1.7 µM doxorubicin (Doxo), or increasing concentrations of E2 (10 nM, 50 nM, and 100 nM) for 16 hr or 48 hr, as indicated. Cleaved and uncleaved poly-ADP-ribose polymerase (PARP) protein expression was analyzed by western blotting. Graphical data points in A, C, D, and E are means ± S.D. of three independent experiments (*** *P*<0.001).

It has been well-documented that E2 stimulates the growth of ER-positive breast cancer cells by promoting the G_1_-to-S-phase transition and that Noxa plays a prominent role in apoptosis. Therefore, to analyze the physiological response of E2-mediated induction of Noxa in ER-positive breast cancer cells, we investigated how loss of Noxa affected cell viability, cell cycle progression, and apoptosis under normal physiological conditions, in the absence of cellular stress. We used three different siRNAs that specifically target Noxa to knock down Noxa mRNA and protein expression in MCF7 cells (Fig. 4A & B). As compared to transfection with non-silencing control (NS) siRNA, transfection with Noxa siRNA caused a more than 50% reduction in cell viability when viability was assayed 48 hr post-transfection (Fig. 4C). Therefore, we did additional experiments to test if the loss of cell viability that we observed when Noxa was knocked down was due to a reduction in the rate of cell cycle progression or an increase in the rate of apoptosis. Interestingly, the results of flow cytometric analysis of propidium iodide (PI)-stained cells showed that knocking down Noxa increases the length of the G_1_/G_0_-phase (Fig. 4D), supporting a role for Noxa as a regulator of the G_1_-to-S phase transition. This raises the possibility that E2-dependent induction of Noxa may be required for E2 to stimulate cell cycle progression. Indeed, E2 treatment induced S-phase entry in MCF7 cells; however, E2-mediated induction of S-phase entry was blocked when Noxa was knocked down (Fig. 4E). Flow-cytometric analysis of the S-phase population using Bromodeoxyuridine (BrdU) incorporation assays showed similar results (data not shown). Notably, although Noxa is typically a pro-apoptotic protein, we found that in the absence of cellular stress, induction of Noxa expression by E2 was associated with cell cycle progression (Fig. 4D & E), but not with apoptosis (Fig. 4F), as assayed by Poly-ADP-Ribose Polymerase (PARP) cleavage using doxorubicin treatment as a positive control. E2 treatment induced Noxa protein expression at 16 hr post-treatment (Fig. 1A) but did not induce PARP cleavage at the same time point (Fig. 4F), or even at later time points (Fig. 4F). Overall, these data suggest that E2-mediated upregulation of Noxa is not sufficient to induce apoptosis under normal, unstressed conditions, but that upregulation of Noxa by E2 is a novel requirement for cell cycle progression in ER-positive breast cancer cells.

## Discussion

Estrogen (E2), as a mitogen, has been well documented to stimulate cell growth and inhibit apoptosis in ER-positive breast cancer cells [36]. Intriguingly, the present study and other recent reports [26], [27] have demonstrated that E2 induces Noxa expression. While Noxa is well-known for its role as a proapoptotic member of the Bcl-2 family, it has become increasingly evident that Noxa plays many roles in other cellular processes, as well. Therefore, we sought to investigate the mechanisms underlying E2-induced Noxa expression and its physiological relevance to ER-positive breast cancer.

First, we used siRNA-mediated protein knockdown approaches to demonstrate that, in the absence of genomic-damaging agents and cellular-stressing agents, E2 induces Noxa expression via p53-independent mechanisms that involve ERα. This observation is strengthened by the fact that while p53 was bound to the *NOXA* promoter in response to treatment with DNA-damaging agents, p53 failed to bind to the *NOXA* promoter in untreated cells and E2-treated cells. However, E2 increased the ability of ERα to bind to the *NOXA* promoter. This is consistent with E2 being able to induce Noxa expression in breast cancer cells, which we report in the present study and which has also been described by another group of investigators [27]. Of note, Noxa expression was found to be co-clustered with ERα expression in breast tumor biopsy specimens [27], suggesting that ERα-dependent upregulation of Noxa is of direct relevance to breast cancer biology. Thus, our studies demonstrate that proproliferative ERα and proapoptotic p53 differentially regulate the function of Noxa in unstressed versus stressed contexts, respectively. Importantly, our data and that of others suggest that Noxa appears to be playing a proproliferative role in a p53-independent manner under certain contexts, while under other contexts, Noxa has a p53-dependent proapoptotic role. Future studies should clarify the mechanisms that regulate the balance between these two opposing functions of Noxa.

Previous reports have demonstrated that E2 induces the expression of Noxa and c-Myc, and that c-Myc regulates Noxa expression [22]; however, detailed mechanisms linking all of these processes together remained to be identified. The data shown in the present study are in agreement with these previous reports, as we provide evidence that E2 induces c-Myc expression which, in turn, increases Noxa expression. Furthermore, we have discovered novel crosstalk between ERα and E2F1/RB signaling pathways in the upregulation of Noxa. It has been shown previously that E2 treatment increases E2F1 protein levels, thereby affecting E2F1-mediated transcription in MCF7 cells [37]. We report that E2F1 mediates an E2-dependent increase in the amount of ERα that is bound to the *NOXA* promoter, resulting in the upregulation of Noxa. Because E2F1 plays a critical role in controlling S-phase entry [38], [39], these findings indicate that Noxa could mediate crosstalk between ERα and E2F1/RB, as regulators of E2-induced cell cycle progression.

Our experiments have, for the first time, revealed a requirement for Noxa expression during cell cycle progression in MCF7 breast cancer cells. Loss of Noxa increases the size of the G_1_/G_0_-phase cell population and decreases that of the S-phase population, suggesting that Noxa expression is required for S-phase entry in unstressed cells. To strengthen the significance of these findings, we have eliminated the likelihood of either mutations occurring within the *NOXA* gene, or alternatively spliced *NOXA* gene products [40] contributing to the proproliferative functions of Noxa reported here (data not shown). Our results showing that Noxa expression is required for S-phase entry in breast cancer cells are further supported by data from another study which showed that Noxa expression is induced during the S-phase in actively dividing B cells [41]. Furthermore, enhanced Noxa expression was associated with prostate cancer progression in a recent report [42], and high expression of Noxa has also been observed in chronic lymphocytic leukemia (CLL) cells [43], suggesting that Noxa likely plays a role in highly proliferating cells of various tissue types. While additional studies are needed to elucidate the mechanisms by which Noxa promotes E2-induced cell cycle progression, it is reasonable to speculate the involvement of phosphorylation of Noxa at serine 13, since it has been shown that glucose-dependent phosphorylation of Noxa at serine 13 promotes cell growth via preferential channeling of glucose to the pentose phosphate pathway (PPP) [17], [18]. An intriguing question is whether a link between estrogen signaling and glucose metabolism exists, which would favor an anabolic state that is conducive to cellular proliferation.

Notably, E2-dependent upregulation of Noxa on its own did not induce apoptosis in our cell culture model under normal, unstressed conditions. These results are consistent with earlier observations that the proapoptotic functions of Noxa are highly restricted and dependent upon cell type and the context of cellular stimuli. For example, when wild type mouse embryonic fibroblasts (MEFs) that had been transduced with *E1A*, *MYC*, and *H-RAS-G12V* oncogenes were subjected to RNA interference to downregulate endogenous Noxa, they were protected from p53-dependent apoptosis, supporting a proapoptotic role for Noxa under these conditions [44]. On the other hand, hematopoietic cells from *NOXA* knockout mice were normally sensitive to apoptosis induction, suggesting that Noxa was dispensable for apoptosis in this context [45]. Collectively, these lines of evidence indicate that Noxa's pro-apoptotic function is highly regulated and cell-type specific. Since cell cycle regulation and apoptosis are closely linked cellular processes, future studies should focus on identifying the cellular signals that differentially activate the proproliferative and proapoptotic functions of Noxa.

To conclude, in addition to the already well-documented proapoptotic function of Noxa, we herein identify a novel role for Noxa as a positive regulator of cell cycle progression in ER-positive breast cancer cells. Our studies suggest that these dual functions of Noxa could have important clinical implications, especially in ER-positive breast cancer patients and in cases where chemotherapeutic and hormonal-therapy drugs, which modulate Noxa expression, are administered. Presumably, in coordination with other proteins, Noxa could participate in balancing cell survival and cell death in a stimuli- and cell-context-dependent manner. Our data suggest that a more detailed understanding of Noxa's many roles as a regulator of diverse cellular functions is required in order to ascertain how Noxa might play a differential role in normal versus tumorigenic tissues.

## Materials and Methods

### Cell lines and culture

The human breast cancer cell line MCF7 from ATCC (Manassas, VA) was maintained in Dulbecco's Modified Eagle's Medium (DMEM) supplemented with 10% fetal bovine serum (FBS) (Invitrogen) at 37°C, under a humidified atmosphere of 5% carbon dioxide. Prior to 17β-estradiol (E2) treatment, cells were cultured in DMEM containing 10% dextran-charcoal-treated FBS for 3 days.

### Small interfering RNA (siRNA) transfection

siRNA at a final concentration of 50 nM was transfected into cells using Lipofectamine 2000 (Invitrogen). At various time points after transfection and treatment, cells were harvested and processed for RNA and protein analysis. The following siRNA were purchased from Dharmacon: Non-silencing control (NS) siRNA (catalogue #D-001206-13-20), ERα siRNA (catalogue #M-003401-04-10), and p53 siRNA (catalogue #M-003329-01-0050). The following siRNA were purchased from Ambion: c-Myc siRNA (catalogue #AM4250), E2F1 siRNA (catalogue #AM16708), and Noxa siRNA (catalogue #16708A). For Noxa siRNA, 3 different siRNA sequences were used alone or in combination, as indicated in the figure legends: S1 (ID #5926); S2 (ID #144355) and S3 (ID #144356).

### Quantitative real-time polymerase chain reaction (qPCR)

qPCR was performed as described previously [46], [47]. Briefly, total RNA was isolated using the Absolutely RNA Miniprep Kit (Stratagene). For analyzing the mRNA expression of different genes, 500 ng of total RNA was reverse transcribed in a 20 µL reaction using the iScript cDNA Synthesis Kit (Bio-Rad). qPCR was carried out in a Prism 7300 Sequence Detection System (Applied Biosystems) using SYBR Green Supermix (Bio-Rad). The relative mRNA levels were calculated using the ΔΔ*Ct* method using endogenous β-actin mRNA as an internal control. The following primer sets were used for qPCR:

Actin forward: 5′-ATG GGT CAG AAG GAT TCC TAT-3′

Actin reverse: 5′-AAG GTC TCA AAC ATG ATC TGG G-3′

NOXA forward: 5′-GCA GAG CTG GAA GTC GAG TGT-3′

NOXA reverse: 5′-CTC TTT TGA AGG AGT CCC CTC AT-3′

pS2 forward: 5′-CGT GAA AGA CAG AAT TGT GGT TTT-3′

pS2 reverse: 5′-CGT CGA AAC AGC AGC CCT TA-3′

NCL forward: 5′-TGC TGC GGA GAT CAG ATT AGT C-3′

NCL reverse: 5′-CAT CGA TCT CTG TTC CCT GCT T-3′

### Cell cycle analysis

MCF7 cells were harvested after siRNA transfection and E2 treatment. Cell cycle distribution was assayed by staining total cellular DNA with propidium iodide (PI). Flow cytometry was performed on a FACScan cytometer (Pharmingen), and the results were analyzed using ModFit software.

### Cell viability assay

The Cell Titer-Glo Assay kit (Promega) was used to assay the number of viable cells by measuring total ATP levels, according to the manufacturer's instructions.

### Chromatin immunoprecipitation (ChIP) assay

ChIP was performed as described previously [46], [47]. Briefly, MCF7 cells were washed once with phosphate buffered saline (PBS) and then crosslinked with 1.5% formaldehyde at 37°C for 10 minutes. After washing with ice cold PBS twice, the cells were collected in lysis buffer (0.5% SDS, 5.6 mM EDTA, 33 mM Tris-HCl, pH 8.1, 0.5% Triton X-100, 84 mM NaCl) and lysed for 30 minutes on ice. Cell lysates were sonicated using a Sonicator 3000 (Misonix, NY) and then diluted 5-fold with dilution buffer (0.01% SDS, 1.2 mM EDTA, 16.7 mM Tris-HCl, pH 8.1, 1.1% Triton X-100, 167 mM NaCl). Diluted cell lysates were pre-cleared with salmon sperm DNA/Protein A agarose (Millipore) for 2 hours at 4°C. Five micrograms of specific antibody or normal IgG were used to immunoprecipitate protein-DNA complexes from pre-cleared supernatants containing 200 µg of protein. Immunoprecipitated DNA was amplified by PCR using AccuPrime TaqDNA Polymerase (Invitrogen), resolved by agarose gel electrophoresis, and visualized with ethidium bromide staining. To analyze protein binding to the *NOXA* promoter, the following primer set was used: forward, 5′-TAC GTC ACC AGG GAA GTT CTC A-3′; reverse, 5′-GGA ACC TCA GCC TCC AAC TG-3′. A control (nonspecific, NS) site within the *NOXA* promoter (at −2 kB) was analyzed using the following primer set: forward, 5′-AGG GTG CGT ATT TGA ACG AC-3′; reverse, 5′-GGC TGA TGT TGG CTG TTT TT-3′. To analyze a potential ERE site (at −3.7 kB), the following primer set was used: forward, 5′-GCT GGA GTG CAA TGG TGT AA-3′; reverse, 5′-CAG TGT GGC TCA CGC TTG TA-3′.

### Co-immunoprecipitation (co-IP) assay

Co-IP assays were performed as described previously [47]. Briefly, MCF7 cells were washed in PBS and harvested in NENT buffer containing a protease inhibitor cocktail (Roche Diagnostics). After lysis for 30 min at 4°C, the whole cell lysate was cleared by centrifugation for 10 min at 14,000 rpm at 4°C. Two mg of lysate was then precleared with protein G/agarose beads (Invitrogen) and subsequently incubated with 6 µg of normal IgG or specific antibody overnight at 4°C. Antibody-bound protein complexes were immunoprecipitated with protein G/agarose, washed 3 times with NENT buffer, boiled in SDS sample buffer, and resolved by SDS-PAGE, followed by western blotting analysis. Protein bands were visualized using the ECL method (Pierce).

### Antibodies

Normal mouse IgG (sc-2025), normal rabbit IgG (sc-2027), and antibodies against Noxa (sc-26917), c-Myc (sc-40), RB (sc-50), E2F1 (sc-193), ERα (sc-543, sc-8005), and p53 (sc-126) were purchased from Santa Cruz Biotechnology. The β-actin antibody (A2066) was purchased from Sigma. The PARP antibody (#9542) was obtained from Cell Signaling. Horseradish peroxidase-conjugated secondary antibodies were obtained from Bio-Rad.

### Statistical analysis

Graphical data values are represented as means of triplicate experiments ± standard deviations. Unpaired Student's t-tests were performed to analyze the differences between control and experimental groups. A p-value less than 0.05 was considered to be statistically significant.
